# ICU nurses’ knowledge, attitude, and practice regarding blood glucose management in critically ill patients: A multicenter cross-sectional study

**DOI:** 10.1097/MD.0000000000046132

**Published:** 2026-05-12

**Authors:** Hongmei Zhang, Zhi Zeng, Sha Xie, Xiuru Yang, Fenglin Yan, Zhenghua Liang, Qiuhong He

**Affiliations:** a Department of Intensive Care Unit, Mianyang Central Hospital, Affiliated with the School of Medicine, University of Electronic Science and Technology of China, Mianyang, China; b School of Nursing, North Sichuan Medical College, Nanchong, China.

**Keywords:** blood glucose, critical care, knowledge, attitude, and practice, nurse

## Abstract

This study investigates the knowledge, attitude, and practice (KAP) of ICU nurses regarding blood glucose management in critically ill patients, providing insights for enhancing the quality of care. A questionnaire survey was conducted among 310 ICU nurses from 5 top-tier general hospitals in Mianyang City, utilizing a general information questionnaire and a specialized survey on blood glucose management in ICU settings. The ICU nurses’ scores in the knowledge, attitude, and practice dimensions of blood glucose management were (32.56 ± 10.58), (49.75 ± 7.41), and (58.83 ± 7.99) points, respectively. The total KAP score was (141.14 ± 21.16), indicating a moderate level. Multiple linear regression analysis identified gender, availability of blood glucose management protocols, participation in blood glucose management training, and whether served as a clinical teacher qualifications as significant factors influencing ICU nurses’ KAP (*P* < .05). There is an urgent need to improve the KAP levels of ICU nurses in blood glucose management for critically ill patients. Hospital administrators should implement targeted training programs tailored to the characteristics of ICU nurses to enhance the quality of blood glucose management and ensure patient safety.

## 1. Introduction

Normal blood glucose levels are important for maintaining the body’s physiological functions. Critically ill patients often develop stress hyperglycemia due to causes such as trauma, shock, and infection, or experience hypoglycemia following insulin therapy.^[1]^ Studies show that the incidence of stress hyperglycemia in critically ill patients ranges between 30% and 80%.^[2]^ Stress hyperglycemia can lead to increased catabolism, poor wound healing, infections, and other complications. It is an independent risk factor associated with prognosis, regardless of whether there has been a previous diagnosis of diabetes.^[3]^ Compared to hyperglycemia, the harm of hypoglycemia to critically ill patients should not be overlooked. Since the brain is highly dependent on glucose for energy, hypoglycemia can lead to irreversible neurological damage and increase the risk of mortality.^[4]^ Reports have shown that the incidence of hypoglycemia in critically ill patients ranges from 18% to 65%, and the mortality rate for patients with severe hypoglycemia is approximately 35.4% to 50.2%.^[5]^ Therefore, strengthening blood glucose management in critically ill patients is of vital importance.

Continuous insulin therapy based on an insulin infusion protocol is the standard approach for controlling blood glucose levels in critically ill adults^[6]^; however, adherence to the protocol in the ICU environment is often suboptimal.^[7]^ On one hand, although guidelines recommend avoiding blood glucose abnormalities (severe hyperglycemia, blood glucose >10 mmol/L [>180 mg/dL] or hypoglycemia, <4.4 mmol/L [<80 mg/dL]), consistent glycemic control remains challenging for critically ill patients who are hemodynamically unstable and have varying medication and nutritional requirements.^[8]^ On the other hand, while ICUs develop their own localized protocols for glycemic control, these protocols often differ in specific operational guidelines and actual effectiveness, which increases the difficulty of implementation.^[2]^

ICU nurses are the direct implementers of blood glucose management for critically ill patients, responsible for insulin infusion and adjustment in insulin infusion protocol, providing physical care, and addressing the concerns of patients’ families.^[9]^ However, 55.5% of healthcare professionals exhibit nonstandard practice during the blood glucose management of critically ill patients, which directly affects the treatment and prognosis of these patients.^[10]^ The knowledge, attitude, and practice (KAP) theory^[11]^ posits that knowledge and attitude directly influence individual behavior, while knowledge also indirectly affects behavior through attitude. The KAP theory is commonly used to develop questionnaires to understand the knowledge, attitude, and practice of a specific population, and the results are utilized to design interventions to improve behavior.^[12]^ Therefore, this study aims to investigate the current status of KAP among ICU nurses regarding blood glucose management in critically ill patients based on the KAP theory, analyze its influencing factors, and provide a reference for clinical nursing managers to develop targeted interventions and enhance the blood glucose management skills of ICU nurses.

## 2. Design and methods

### 2.1. Design

This study was designed and conducted as a cross-sectional study, and the reporting of this study was done following the STROBE statement. We hypothesize that these findings may help scholars identify KAP gaps in the blood glucose management of critically ill patients, and the results may provide a reliable basis for improving blood glucose management in critically ill patients. The specific objectives include: First, to understand ICU nurses’ knowledge regarding blood glucose management in critically ill patients; Second, to understand ICU nurses’ attitude towards blood glucose management in critically ill patients; Third, to understand ICU nurses’ practice in blood glucose management for critically ill patients; and Fourth, to identify various factors affecting the KAP level of ICU nurses regarding blood glucose management in critically ill patients.

### 2.2. Participants

This survey was conducted from November 1 to February 28, 2024. A convenience sampling method was used to select adult ICU nurses from 5 general hospitals in Mianyang City, China, to complete the survey. Inclusion criteria were as follows: Registered nurses in China; ≥1 year of ICU clinical nursing experience; Voluntary participation in this study. Exclusion criteria included: Nurses who were not on duty during the survey period (e.g., sick leave, maternity leave, etc); Internship, training, and advanced practice nurses. The sample size was calculated using Kendall method, which recommends 5 to 10 times the number of variables for adequate statistical power.^[13]^ This study considered 9 demographic factors and 39 scale items as independent variables. Accounting for a 20% attrition rate, the minimum required sample size was (9 + 39) × 5 × (100% + 20%) = 288 participants.

### 2.3. Survey instruments

#### 2.3.1. General information

The survey included both demographic information and questions regarding blood sugar management training experiences and knowledge needs. It was designed in-house by the research team. The demographic information encompassed age, gender, marital status, education, title, and years of ICU experience. Additionally, the survey covered blood glucose management implementation standards and training experiences.

#### 2.3.2. KAP questionnaire for glucose management

A questionnaire was developed by Jiafeng Xue^[14]^ to assess ICU nurses’ glycemic management of critically ill patients. The content covers 3 dimensions: knowledge, attitude, and practice, with a total of 39 entries.

1. The knowledge dimension consists of 12 entries regarding blood glucose target values, the definition of hypoglycemia, measures to deal with it, and methods of insulin administration, timing, and preservation. Each entry is worth 5 points for a correct answer and 0 points for an incorrect answer, with a score range of 0 to 60 points.
2. The attitude dimension includes 12 items, such as the importance of blood glucose management, enthusiasm for understanding blood glucose management, the importance of training in blood glucose management, and the necessity of developing standardized protocols for blood glucose management.These are measured using a 5-point Likert scale, with each entry scored from 1 (“completely disagree”) to 5 (“completely agree”), for a total score range of 12 to 60 points.
3. The practice dimension includes 15 entries on enteral nutrition management, management of hyperglycemia, frequency of glucose monitoring, insulin regulation, and management of hypoglycemia. These are also measured using a 5-point Likert scale, with scores ranging from 1 (“never”) to 5 (“always”), for a total score range of 15 to 75 points.

The total score of the questionnaire is the sum of the scores from the knowledge, attitude, and practice dimensions, with a range of 39 to 195. Higher scores indicate a better level of nurses’ knowledge of blood glucose management. The score rate for the total questionnaire and each dimension was calculated as the mean score divided by the total possible score of the dimension, multiplied by 100%. Scores >85% were considered high level, <60% as low level, and 60% to 85% as medium level. We obtained permission to use the questionnaire from its authors before the survey. Prior to the formal investigation, 15 ICU nurses were selected for an initial survey. The total Cronbach alpha coefficient of the questionnaire was 0.932, with coefficients of 0.933, 0.945, and 0.927 for the knowledge, attitude, and practice dimensions, respectively.

#### 2.3.3. Data collection methods

Prior to the survey research, team members first contacted the head nurse of the intensive care unit of each target hospital through the social media platform WeChat. The purpose and content of the investigation were communicated, and ICU nurse managers at each hospital were invited to provide target participants who met the inclusion and exclusion criteria. Electronic questionnaires were distributed and returned using the Questionnaire Star platform. An informed consent form is located on the first page of the electronic questionnaire. Respondents who agree to participate in this survey are required to check the informed consent box before proceeding to the formal survey to begin answering questions. All questionnaires are set to be filled out anonymously, and all options are mandatory to ensure that the questionnaire is completed in full before submission. To prevent duplication, the system is configured to allow only 1 submission per IP address.

#### 2.3.4. Statistical analysis

SPSS 27.0 software was used for statistical analysis. Measures conforming to normal distribution are expressed as mean ± standard deviation. Comparisons between groups were made using independent samples *t* tests. Comparisons between multiple groups were analyzed by one-way ANOVA; Linear regression was used to analyze the factors affecting the level of knowledge, belief, and behavior of ICU nursing staff in blood glucose management. Statistical significance was set at *P* < .05.

#### 2.3.5. Ethical and institutional approvals

The study protocol was approved by the Ethics Committee of Mianyang Central Hospital (approval no. S202403171-02). All participants were informed of the study’s purpose and assured of the confidentiality of their personal information. They were also informed of their right to withdraw from the study at any time without any consequences.

## 3. Results

### 3.1. KAP levels of ICU nurses regarding blood glucose management in critically ill patients

**Figure 1.**
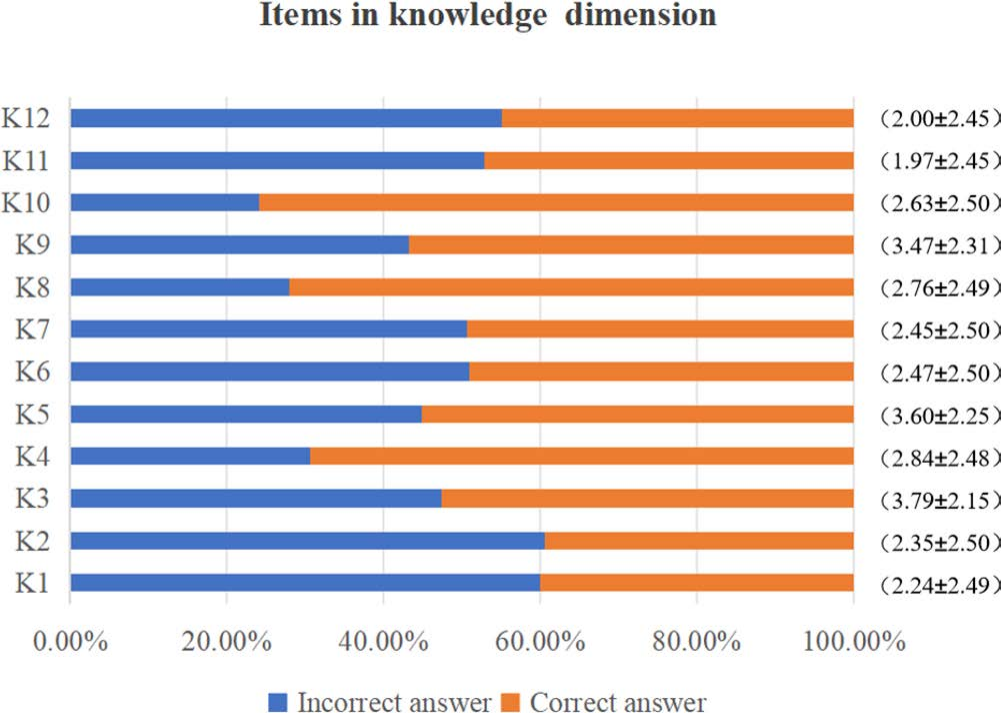
Score rate and average score of each item in the knowledge dimension of ICU nurses’ blood glucose management.

**Figure 2.**
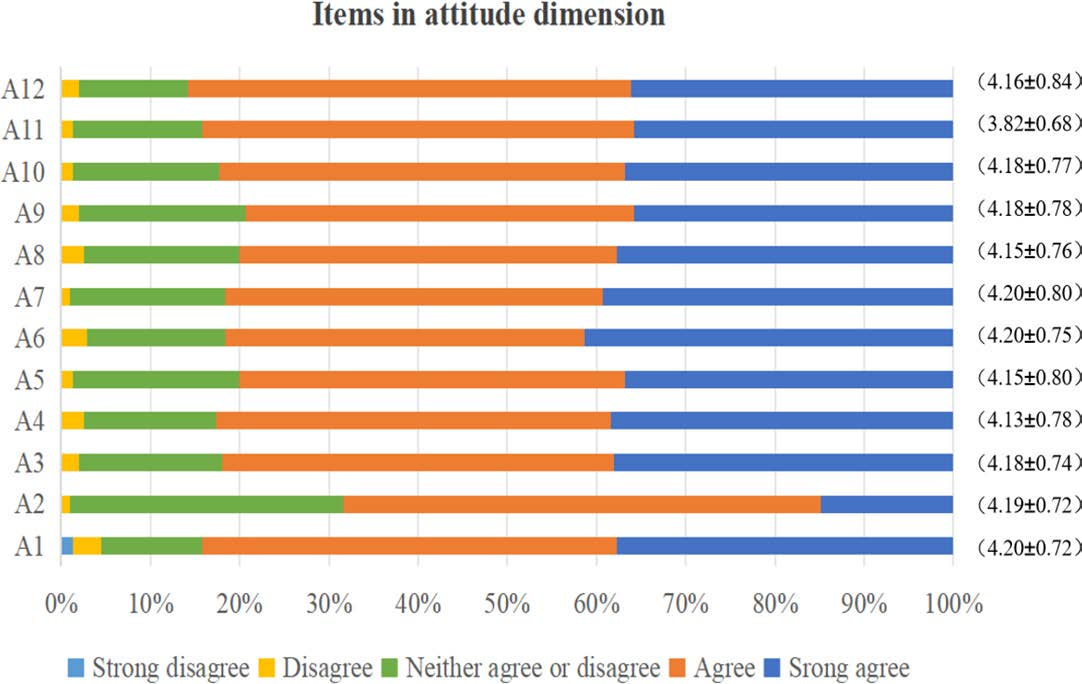
Score rate and average score of each item in the attitude dimension of ICU nurses’ blood glucose management.

**Figure 3.**
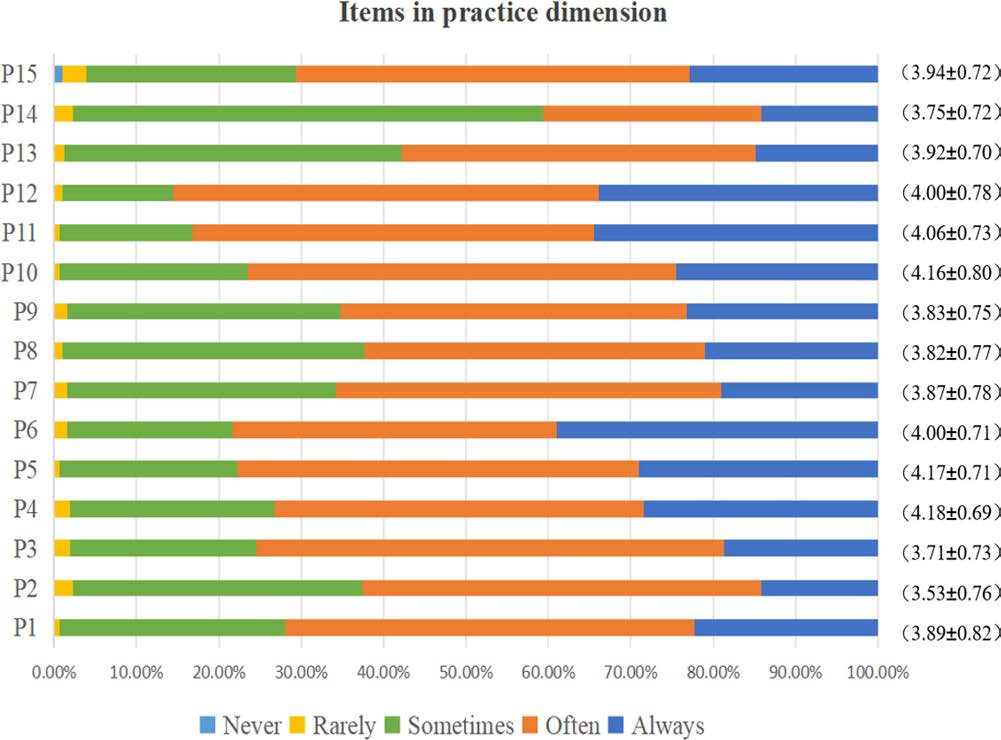
Score rate and average score of each item in the practice dimension of ICU nurses’ blood glucose management.

Three hundred ten ICU nurses scored (32.56 ± 10.58) on the knowledge dimension, (49.75 ± 7.41) on the attitude dimension, (58.83 ± 7.99) on the practice dimension, and (141.14 ± 21.16) on the total scale score of the blood glucose management KAP Survey Scale. The overall KAP of ICU nurses regarding blood glucose management was moderate, as shown in Table 1. Figure 1 presents the scoring rates and average scores for each item in the knowledge dimension of blood glucose management among 310 ICU nurses. Similarly, Figure 2 displays this information for the attitude dimension, while Figure 3 illustrates the corresponding data for the practice dimension (for specific details regarding each item, see Supplementary material 1, Supplemental Digital Content, https://links.lww.com/MD/Q801).

**Table 1:** ICU nurses’ KAP survey scores for blood glucose management (n = 310).

Project	Score status $\left(\bar{x}\pm s\right)$	Score rate (%)
Knowledge dimension	32.56 ± 10.58	54.26%
Attitude dimension	49.75 ± 7.41	82.91%
Practice dimension	58.83 ± 7.99	78.44%
Total score of the KAP	141.14 ± 21.16	72.37%

Score rate 1 actual score/theoretical maximum score × 100%.

### 3.2. Comparison of KAP scale scores among ICU nurses with different characteristics in blood glucose management for critically ill patients

A one-way analysis of variance revealed statistically significant differences in ICU nurses’ Knowledge and Trust Behavior Scale scores based on gender, age, marital status, years of work experience, education, title, blood glucose management implementation standards, blood glucose management training experience, and clinical teaching qualifications (*P* < .05), as shown in Table 2.

**Table 2:** Comparison of ICU nurses’ KAP scores for glucose management by different characteristics $\left(\bar{x}\pm s\right)$.

Project	Categorization	Number	Total score of the questionnaire		
			Score	Statistical value	*P*-value
Gender	Male	58	133.00 ± 23.14	-3.039	.003
	Female	252	143.02 ± 20.27		
Age	20–30 years old	194	136.75 ± 22.67	12.076	<.001
	31–40 years old	107	148.76 ± 16.22		
	41–50 years old	9	145.22 ± 12.43		
Marital status	Unmarried	136	134.69 ± 22.90	-4.786	<.001
	Married	174	146.18 ± 18.24		
Working experience	<5 yr	159	136.39 ± 22.12	7.948	<.001
	5–10 yr	89	143.37 ± 21.74		
	11–15 yr	50	152.06 ± 12.66		
	>15 yr	12	142.08 ± 12.06		
Educational attainment	Specialized subject	65	121.03 ± 15.57	-9.873	<.001
	Undergraduate	245	146.48 ± 19.16		
Title	Nurses	98	136.64 ± 22.46	6.009	<.001
	Physiotherapists	108	138.56 ± 22.69		
	Nurse practitioner-in-charge	98	147.94 ± 16.38		
	Associate Chief Nurse	6	150.00 ± 11.42		
Availability of blood glucose management protocols	No	43	119.44 ± 22.25	-7.939	<.001
	Yes	267	144.64 ± 18.81		
Participation in blood glucose management training	No	69	113.86 ± 13.28	-16.779	<.001
	Yes	241	148.95 ± 15.85		
Experience as a clinical teacher	No	73	129.56 ± 18.22	-7.774	<.001
	Yes	236	147.42 ± 20.00		

### 3.3. Multiple linear regression analysis of factors influencing ICU nurses’ KAP regarding blood glucose management in critically ill patients

ICU nurses’ blood glucose management knowledge and belief scores were used as the dependent variables. Factors with statistically significant differences in the results of the univariate analysis were used as independent variables and included in the multiple linear regression analysis. The results indicate that gender, blood glucose management implementation standards, blood glucose management training, and clinical teaching qualifications were influential factors affecting the current status of ICU nurses’ knowledge, beliefs, and behaviors in blood glucose management (*P* < .05). These factors explained 56.5% of the total variation (*F* = 45.589, *P* < .001; *R*^2^ = 0.578, adjusted *R*^2^ = 0.565). The results of the regression analysis are presented in Table 3.

**Table 3:** Multiple linear regression analysis of factors influencing the level of KAP of ICU nurses in blood glucose management.

	*B*	SE	Beta	*t*	*P*	95% CI	
						Lower limit	Upper limit
(Constant)	97.315	9.15		10.636	<.01	79.309	115.321
Gender	5.302	2.109	0.098	2.514	.012	1.152	9.452
Age	-2.616	2.64	-0.068	-0.991	.322	-7.812	2.579
Marital status	3.203	2.288	0.075	1.4	.163	-1.3	7.706
Working years	1.498	1.31	0.062	1.144	.254	-1.079	4.075
Highest degree	-4.882	3.434	-0.094	-1.422	.156	-11.639	1.876
The title of a professional post	0.666	1.916	0.027	0.348	.728	-3.105	4.437
Availability of blood glucose management protocols	17.519	2.513	0.287	6.971	<.01	12.573	22.464
Participation in blood glucose management training	28.972	3.328	0.57	8.707	<.01	22.424	35.521
Experience as a clinical teacher	7.635	2.042	0.173	3.74	<.01	3.618	11.653

*R*^2^ = 0.578; after the adjustment *R*^2^ = 0.565; *F* = 45.589, *P* < .001.

## 4. Discussion

### 4.1. Current state of KAP among ICU nurses in blood glucose management for critically ill patients

The survey results indicate that the total KAP score for ICU nurses in managing blood glucose among critically ill patients was (141.14 ± 21.16), with a scoring rate of 72.37%. The dimension scores, ranked from highest to lowest, were: attitude dimension (49.75 ± 7.41), scoring rate 82.91%; practice dimension (58.83 ± 7.99), scoring rate 78.44%; and knowledge dimension (32.56 ± 10.58), scoring rate 54.26%. Overall, the KAP level of ICU nurses regarding blood glucose management in critically ill patients was moderate, which aligns with the findings reported by Roco et al,^[15]^ further supporting the view that the blood glucose management level of ICU nurses needs urgent improvement. Notably, the score in the knowledge dimension was significantly lower than that in the national survey by Huang et al,^[16]^ which may be attributed to the fact that the study sample was drawn from inland China, reflecting a certain lag in knowledge updating and clinical practice transformation in this region. Therefore, there is an urgent need to establish systematic and targeted training mechanisms to promote the effective transformation of knowledge into clinical competence, thereby narrowing the development gap between regions and ensuring that nursing quality develops in sync with national standards.

In terms of knowledge, the score rate of ICU nurses was only 54.26%, which was at a low level. The higher score rates were for knowing the interval of retesting blood glucose after hypoglycemia treatment and for knowing that patients should use a 50% glucose injection for intravenous injection when hypoglycemia occurs. This indicates that ICU nurses have a good grasp of the knowledge of insulin use and hypoglycemia management, which may be related to the fact that these contents are more common and operable in daily work. However, only 40% of nurses knew that when using a glucose meter to measure blood glucose, the blood glucose level would be high due to hard squeezing during blood collection, and 60.6% of nurses did not know the optimal blood glucose target for severely ill patients. The lack of mastery of this knowledge may be related to the focus of the training content. Some nurses may pay more attention to practical operations and ignore the learning of theoretical knowledge.

In terms of attitude, the score rate of ICU nurses was 82.91%, which was at a high level. The vast majority of nurses believe that it is necessary for the department to supervise blood glucose management and that it is necessary to strengthen the training of ICU nurses on blood glucose management knowledge for critically ill patients. These results indicate that ICU nurses pay more attention to blood glucose management and are willing to learn and participate in it. This positive attitude may be related to the working environment of ICU nurses. The condition of ICU patients is complex and rapidly changing, and blood glucose management has a direct impact on the prognosis of patients, so ICU nurses pay more attention to it.

In terms of practice, the blood glucose management behavior score rate of ICU nurses was 78.44%, which was at a middle level. Most of the nurses can perform blood glucose management measures well in practice; for example, 85.81% of the nurses will judge the insulin sensitivity of the patients when giving insulin treatment, and 85.48% of the nurses will remeasure blood glucose 15 to 30 minutes after hypoglycemia treatment. These results suggest that the behavior of ICU nurses regarding insulin use and hypoglycemia management is relatively standardized. However, only 40.65% of nurses measured blood glucose when changing the rate of intra- and parenteral nutrition intake. The lack of implementation of these behaviors may be related to the high work intensity of nurses, the lack of familiarity with operation procedures, or the lack of knowledge due to insufficient training.^[17]^

### 4.2. Determinants of KAP levels among ICU nurses in blood glucose management for critically ill patients

The results of this KAP survey show that gender, availability of blood glucose management protocols, participation in blood glucose management training, and status as a clinical teaching teacher are factors influencing the KAP levels of ICU nurses in blood glucose management (*P* < .05). The analysis is as follows: Gender: Female ICU nurses demonstrated higher KAP levels in blood glucose management than male ICU nurses, which is consistent with the findings of Cui Peirong et al.^[18]^ The reason may be that male nurses have poorer professional identity, which hinders their theoretical knowledge improvement.^[19]^ This suggests that managers should give more attention to male nurses during training, and experienced female nurses could guide male nurses through team collaboration to achieve knowledge sharing and promote quality improvement.^[20]^ Availability of blood glucose management protocols: Establishing systematic blood glucose management protocols is an important measure to standardize clinical practice and reduce arbitrary nursing decisions.^[21]^ It is recommended that medical institutions develop scientific and standardized blood glucose management protocols based on evidence-based medicine and international authoritative guidelines, combined with national clinical practice characteristics.^[22]^ The implementation of these standards should be incorporated into the nursing quality evaluation system, using a multi-dimensional assessment approach that combines process monitoring with outcome evaluation, regularly evaluating implementation effectiveness to ensure effective execution and continuous improvement, thereby enhancing the scientific nature and standardization of blood glucose management. Participation in blood glucose management training: Previous studies have shown that systematic training can effectively improve nurses’ theoretical knowledge and practical skills in blood glucose management, thereby increasing the rate of achieving target blood glucose control in patients. It is recommended that medical institutions include blood glucose management training as a mandatory course in the continuing education of ICU nurses, adopting innovative teaching methods such as stratified training^[23]^ and simulated scenario teaching^[24]^ to enhance training effectiveness. Meanwhile, a training effectiveness tracking mechanism should be established to regularly evaluate nurses’ knowledge mastery and clinical application abilities, ensuring that training outcomes are effectively transformed into clinical practice. Whether served as a clinical teacher: In China, clinical teachers are typically senior nurses with extensive clinical practice experience, whose long-term practical accumulation has given them an in-depth understanding of blood glucose management standards. Meanwhile, teaching responsibilities prompt teachers to continuously update their professional knowledge and further consolidate and deepen their own knowledge systems through teaching interactions. Therefore, it is recommended that clinical nursing managers should focus on the rational pairing of senior and junior nurses in staffing and scheduling arrangements to establish an effective mentorship mechanism. Additionally, academic exchange and external learning opportunities should be provided to junior ICU nurses to gradually narrow the capability gap between nurses of different seniority levels, thereby enhancing overall blood glucose management and providing patients with higher quality medical services.^[25]^

### 4.3. Limitation

This study has the following limitations: First, the study adopted a convenience sampling method, which is prone to introducing regional bias. Due to significant differences in medical resource allocation and nursing practices across different regions, the generalizability and representativeness of the findings are somewhat limited. It is recommended that future studies expand the sample coverage to include medical institutions from more diverse regions to enhance the external validity of the research. Second, the questionnaire was self-administered, and the self-reported content by nurses is susceptible to recall bias and social desirability bias, although the study has controlled for these biases through anonymous and confidential measures. Future research could consider employing mixed research methods supplemented by objective measurement indicators or behavioral observation to improve the accuracy and reliability of the data. Finally, future studies should further explore the long-term impact of comprehensive depression management training on medical students’ clinical practice behaviors and patient outcomes, providing more targeted and guiding evidence for clinical practice.

## 5. Summary

ICU nurses’ knowledge level and practical skills regarding blood glucose management in critically ill patients need improvement, but their attitudes are relatively positive. Gender, availability of blood glucose management protocols, participation in blood glucose management training, and whether served as a clinical teacher may be factors influencing ICU nurses’ KAP level regarding blood glucose management in critically ill patients. Nursing managers should emphasize relevant theoretical knowledge and practical skills training for ICU nurses to improve their blood glucose management level, thereby enhancing patient outcomes.

## Acknowledgments

The authors are very grateful to all the ICU nurses who participated in this study.

## Author contributions

**Conceptualization**: Hongmei Zhang, Zhi Zeng, Qiuhong He.

**Data curation**: Zhi Zeng, Sha Xie, Xiuru Yang, Fenglin Yan, Zhenghua Liang.

**Writing – original draft**: Hongmei Zhang, Zhi Zeng.

**Writing – review & editing**: Qiuhong He.

## Supplementary Material
