# Supplementary material for: ICU nurses’ knowledge, attitude, and practice regarding blood glucose management in critically ill patients: A multicenter cross-sectional study
Source: Medicine (Baltimore). 2026 May 12;104(49):e46132. doi: 10.1097/MD.0000000000046132 (PMC12688723; doi:10.1097/MD.0000000000046132)
Supplement: Supplementary file 1 [file medi-104-e46132-s001.docx]

Supplementary Material 1 KAP Questionnaire for Glucose Management

| **Dimension** | **Item** |
| --- | --- |
| **Knowledge Dimension** | After managing the patient’s hypoglycemia, blood glucose should be retested at 15-minute intervals. |
|  | Optimal management of hypoglycemia in patients: 50% dextrose injection IV push. |
|  | Continuous IV pumping is the best way to use insulin to control blood glucose in critically ill patients. |
|  | When pumping insulin to regulate blood glucose intravenously, the appropriate concentration to use is 1 U/ml. |
|  | Patients can have higher than target blood glucose values with enteral/parenteral nutrition or oral feeding. |
|  | Glucose values for optimal glucose targets in critically ill patients: randomized glucose values. |
|  | Critically ill patients with blood glucose less than 2.8 mmol/L are considered to have experienced hypoglycemia. |
|  | Guidelines recommend initiating insulin therapy in critically ill patients with sustained glucose values ≥10 mmol/L. |
|  | The principle of glycemic control in critically ill patients is to control hyperglycemia and avoid hypoglycemia. |
|  | Opened insulin should be stored in a cool, dry place at room temperature. |
|  | When using a blood glucose meter to measure blood glucose, squeezing hard when collecting blood can cause high blood glucose values. |
|  | The optimal glucose target for critically ill patients is 6.1-7.8 mmol/L. |
| **Attitude dimension** | Do you think it is necessary for the department to monitor blood glucose management? |
|  | Do you believe that there is a need to strengthen the training of ICU nurses in the knowledge of blood glucose management in critically ill patients? |
|  | Do you think it is necessary to implement glucose management for critically ill patients? |
|  | Do you think ICU nurses should know about glycemic management in critically ill patients? |
|  | Do you think there is a need to focus on glycemic management in critically ill patients? |
|  | Do you think there is a need to develop norms for blood glucose management in critically ill patients? |
|  | You are willing to learn about glucose management in critically ill patients. |
|  | Do you think that blood glucose management protocols for critically ill patients should be discussed and developed by healthcare professionals? |
|  | Do you think it is necessary to follow the procedure to test your blood glucose in order to obtain an accurate blood glucose value? |
|  | Your willingness to participate in the management of blood glucose in critically ill patients. |
|  | Do you believe that keeping critically ill patients' blood glucose within the target range is important for patient prognosis? |
|  | What you think you currently know about blood glucose management is good enough to manage your blood glucose? |
| **Practice dimension** | You will determine a patient's insulin sensitivity when giving them insulin therapy. |
|  | You will retest your blood glucose 15-30 minutes after treatment of hypoglycemia. |
|  | You would treat patients with blood glucose below 3.9 mmol/L as hypoglycemic patients. |
|  | You will regulate your insulin dosage according to the standardized protocol for insulin regulation. |
|  | You will follow the procedure for blood glucose testing to ensure that you get accurate blood glucose values. |
|  | You will add a blood glucose test to the patient's blood evolution treatment. |
|  | You would activate insulin when the patient's blood glucose is above 10.0 mmol/L twice in a row. |
|  | You will replenish enteral/parenteral nutrients at an even rate, avoiding large amounts of carbohydrates in a short period of time. |
|  | You will proactively review literature, books, and other materials related to glycemic management in critically ill patients. |
|  | You will value the importance of blood sugar fluctuations in critically ill patients. |
|  | You will add blood glucose measurements when a patient's condition changes. |
|  | You will anticipate and monitor blood glucose changes when administering medications that affect blood glucose. |
|  | You will add a blood glucose test when the patient suddenly develops severe diarrhea. |
|  | You communicate with your colleagues about glucose management in critically ill patients in your day-to-day job. |
|  | You will add blood glucose measurement when changing the rate of enteral and parenteral nutritional intake in patients. |
